# Recommendations for the Development and Implementation of a Residents as Teachers Curriculum

**DOI:** 10.7759/cureus.3053

**Published:** 2018-07-26

**Authors:** Anne Messman, Sara M Kryzaniak, Sylvia Alden, Michael J Pasirstein, Teresa M Chan

**Affiliations:** 1 Department of Emergency Medicine, Wayne State University, Detroit, USA; 2 Department of Emergency Medicine, University of Illinois College of Medicine, Peoria, USA; 3 Department of Emergency Medicine, University of New Mexico, Albuquerque, USA; 4 Department of Emergency Medicine, Drexel University College of Medicine, Philadelphia, USA; 5 Faculty of Health Sciences, McMaster University, Hamilton, CAN

**Keywords:** curriculum development, graduate medical education, medical education, resident as teacher

## Abstract

Teaching junior residents, medical students and other hospital staff is an essential component of a resident physician’s job. While resident physicians provide much of the teaching for their junior learners, few residents are provided with formal training on how to teach effectively. Although some Residents as Teachers (RAT) curricula do exist, there is no consensus on how the curriculum should be administered, content it should include, and how to assess its effectiveness. We seek to provide guidelines and recommendations applicable to any residency program seeking to begin or improve upon a RAT curriculum and provide suggestions on how to assess its effectiveness.

## Introduction and background

Residents are frequently asked to participate in the education of their peers and junior learners. Their role as clinical educators is critical, and residents have been identified by learners as providing a substantial proportion of their teaching [1]. In addition, regulating bodies such as the Accreditation Council on Graduate Medical Education (ACGME) and Liaison Committee on Medical Education (LCME) require that residents assume teaching roles [2,3].

Developing residents’ teaching skills is imperative, however, most medical schools do not provide training on this skill to their students, leaving residents unprepared to assume the role of clinical teacher upon entering residency [4]. There is also significant variability in the education residents receive to train them to become competent teachers. There is no standardized curriculum available, and many programs do not offer Residents as Teachers (RAT) curriculum [5,6]. The impact of this gap in resident education is noted by residents who report feeling unprepared for a successful academic career [7].

Our group aimed to identify the critical components of RAT curricula in order to provide evidence-based recommendations to residencies wishing to create or improve upon existing RAT curricula.

## Review

We sought first to review the existing literature to find best practices regarding RAT curricula. Our literature search included PubMed, MedEdPORTAL, JETem, and Google Scholar, with "resident as teacher" as the search term. Sources referenced in primary articles were also used when relevant. Our investigation included RAT curricula from all medical specialties and was not restricted to emergency medicine (EM). We also studied Medical Student as Teacher curricula when pertinent. Many curricula were identified, but we found no standard, agreed-upon recommendations on how a RAT curriculum should be set up. Upon review of papers that examined the effectiveness of RAT curricula, it is important to note that RAT curricula were found to improve a resident’s teaching skills, even in a short amount of time [8]. Additionally, studies found that RAT curricula improved a resident’s teaching skills regardless of whether participation was mandatory or voluntary [9]. We aim to provide a clear roadmap on how an EM residency could begin and maintain a RAT curriculum, and how to assess its effectiveness.

Platform

There are many options for how a RAT curriculum can be disseminated to the resident. Modalities for RAT curricula include workshops, didactic sessions, lectures, asynchronous modules, seminars and many others. These can be offered either live or online. Many types of curricula take a multi-modal approach and utilize several instructional modalities [4-6, 8-11, 15-19]. In general, the curricula were successful regardless of the modality utilized to communicate the information. Residency programs therefore should utilize the modality that best suits their program and learners. If there is wide faculty interest and lecture time is available, small group workshops may be the best modality. If lecture time is less ample or faculty involvement less robust, asynchronous learning via online modules may be the best option for a program.

It is important to note that there are pre-existing resources and that when starting a RAT curriculum, not all resources need to be manufactured de novo. Several programs have made their curricula publicly available. Please see Table 1 for a list of these resources.

**Table 1:** Open-access residents as teachers curricula.

EMRA Handbook	The EMRA handbook is a PDF which provides some basic descriptions of teaching techniques and conceptual frameworks useful to beginning resident teachers. https://www.emra.org/uploadedfiles/emra/emra_publications/emra-2013residentaseducator-interactive.pdf
University of Virginia School of Medicine	This is a nice general curriculum which includes common mistakes and resident teaching cases. https://www.med-ed.virginia.edu/courses/resasteachers/
University of Alabama at Birmingham School of Medicine	This is an extensive Residents as Teachers curriculum complete with references for further reading. https://www.uab.edu/medicine/home/residents-fellows/current/cert
Academic Life in Emergency Medicine	This resource provides a ready-to-use curriculum for a one-month teaching elective as well as a Prezi presentation regarding teaching skills and an evaluation form to assess the resident teachers. https://www.aliem.com/2016/idea-series-asynchronous-curriculum-for-resident-as-teacher
University of Nevada, Reno School of Medicine	This resource provides teaching modules in the form of slide presentations and includes a module on presentation skills. http://med.unr.edu/gme/current-residents/rats

Timing

RAT curricula need not require a great deal of time to be effective. One study found that a six-hour curriculum showed significant improvement in residents’ teacher ratings three years after the intervention [10]. Comfort and motivation with teaching can be increased after as little as two hours [9]. In a survey study of existing EM RAT curricula, most (72.4%) were administered in the third year of residency and 71.4% were administered within a one-month time period [5].

Teaching is a skill; it has been well documented that skills such as chest compressions in cardiopulmonary resuscitation decay if not reviewed and refreshed periodically [12, 13]. Although this has not been explicitly studied in the literature, correlation would suggest that a successful RAT curriculum should be longitudinal in nature rather than a one-time seminar or conference. To this end, we would recommend that a RAT curriculum include quarterly or semi-annual sessions in order to reduce skill decay and reinforce the material. This should include periodic assessment of skills, which is outlined later in this article.

Curriculum

It is important to start with an overview on why learning to teach is an important skill for residents. Residents serve as an important component of medical education and many will continue to participate in the supervision of learners after graduation. Subsequently, an overview of adult learning theory creates a foundation for more practical lessons on how to teach.

After review of the articles by the author, recurring themes emerged and were confirmed by consensus of the authors regarding topics that should be included in a RAT curriculum. These educational topics can be divided into three main categories: teaching methods, how to supervise junior learners, and advanced educational leadership skills. Examples of how a resident can achieve competency in these three categories can be found in Table 2.

**Table 2:** Curriculum components for a Residents as Teachers (RAT) curriculum. SPIT: Serious, Probable, Interesting, Treatable SNAPPS: Summarize, Narrow, Analyze, Probe, Plan, Select 5Cs: Communication, Cultures, Connections, Comparisons, Communities PIQUED: Preparation, Identification, Questions, Urgency, Educational Modifications, Debrief RAPID: Resuscitation, Analgesia, Patient needs, Intervention, Disposition

Teaching Methods	Clinical Supervision of Junior Learners in the Emergency Department	Educational Leadership Skills
Case-based teaching (SPIT, SNAPPS, Aunt Minnie, Questioning) [14-16]	Orienting learners	Leading teams
Bedside teaching	Communication skills	Role modeling
One-minute preceptor, also known as 5-Step Microskill [17]	Presentation skills	Time management
Procedural skill teaching	Teaching consultation skills (5Cs, PIQUED) [18-20]	Assessment and evaluation
Small group teaching	RAPID mnemonic [21]	Curriculum design
Large group teaching	Giving feedback	Simulation

The teaching methods provide residents with skills to make the most of opportunities to teach learners in a variety of contexts. Supervision of junior learners includes more than imparting medical knowledge; involving students into teams, teaching them to consult colleagues effectively, giving feedback and developing communication skills are also important. Finally, learning about more advanced educational skills benefits residents with an interest in education and provides all residents with a better understanding of the educational system of which they are a part.

Assessment

**Figure 1:**
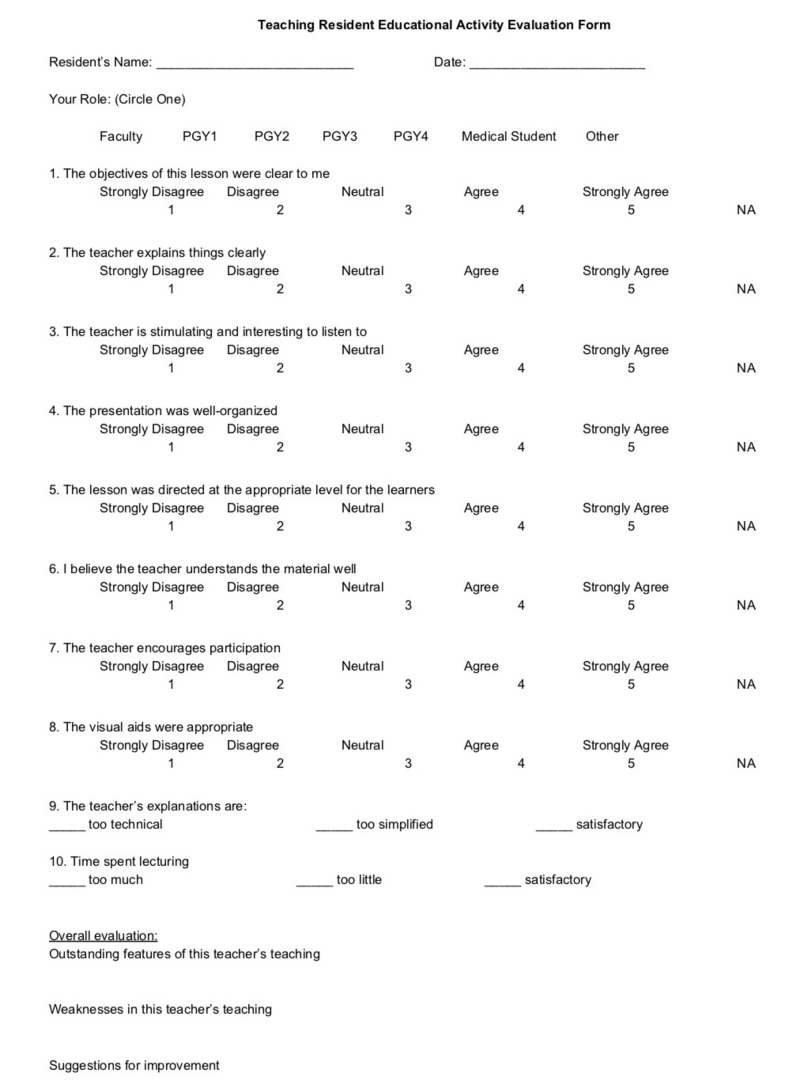
Resident teacher educational activity evaluation form. PGY: Post-Graduate Year NA: Not Applicable

High-quality resident teachers are important for the success of junior residents and medical students, and the use of a formalized assessment tool of resident educators has been demonstrated to be valuable to both the learner and educator [22]. Numerous assessment tools were previously identified by Coverdale et al., and can be compared based on the number of questions, validity, reliability, who performs the rating, and the areas evaluated [23]. For example, both the Stanford Faculty Development Program (http://sfdc.stanford.edu) and the Wisconsin Inventory of Clinical Teaching have been validated and utilize the learners to rate the teachers [24, 25]. The resident’s assessment of their own teaching ability may also be useful information to gather and authors have provided samples of surveys they have used for this purpose [26]. Additionally, the Observed Standard Teaching Evaluation (OSTE) has been validated and uses faculty to rate the teachers using standardized cases and patients/actors [27]. Zabar et al. utilized the OSTE, and in addition to having faculty rate the residents, the residents rated themselves [22]. Ricciotti provides a validated OSTE used in their Obstetrics and Gynecology residency that could be adapted to suit other specialties [28]. Gathering data about the resident’s actual effectiveness as a teacher is also important to gather. Academic Life in Emergency Medicine (ALiEM) provides an evaluation form that can be given to the resident’s learners to gather data about their teaching abilities and is provided in Figure 1.

With such variety in assessment tools, each program will need to individually determine the factors it feels are most important. In Coverdale et al., the number of questions asked range from one to 58, and only eight of the 11 assessment tools identified were found to be valid and reliable [23]. Topics examined include learning climate, promoting understanding and retention, teaching effectiveness, instructor knowledge, role of teachers, and improvement of clinical teaching. One of the easiest methods is to have the learners rate the educators. However, utilizing experienced faculty can offer insight and strategies for teaching improvement. A significant limitation of using faculty to evaluate resident teachers is cost. Morrison et al. noted their initial start-up cost was $454 per resident, whereas in their second year of the study, the cost improved to $120 per resident [27]. These costs do not include institutional contributions of space and faculty release time. Part of the initial fees included a consultant who specialized in medical education, which was only for the first year [28]. Another challenge is that bias can occur if the faculty involved in an OSTE have worked clinically with a resident, as perceptions can be perpetuated. One strategy to help mitigate this effect is to use a standard list of check-boxes for faculty evaluations. Another can include using videos and having multiple faculty rate the teachers, but that can be costly.

As teaching strategies evolve with technology and generational propensity, assessing the educator will need to evolve as well. In the example survey from Johnson et al., the first task is to state the objectives. Additional metrics appear flat and uninspiring, such as asking for a rating on “having taught my learners something new about pediatrics by the end of the rotation [26].” As teachers are using less slide-show based lectures, and more hands-on simulation, the tools used will need to ask the right questions to provide the best feedback to the educator, and can therefore modify the curriculum to better fit the needs of the learners.

## Conclusions

During post-graduate training, residents serve as educators as well as learners. As residents complete their training, many will continue to teach in some capacity. It is an ACGME requirement that residents teach their learners and an LCME requirement that residents teach medical students. Despite these requirements, there is large variability in the amount of training that residents receive in order to become competent teachers. We suggest here a roadmap that could be easily implemented by any residency program wishing to start or maintain a RAT curriculum, and provide recommendations on how a program’s RAT curriculum can be assessed to ensure it is effective.
